# Needle length in pines as a key trait regulating hydraulic resistance

**DOI:** 10.1093/aob/mcaf174

**Published:** 2025-07-29

**Authors:** Giovanni Bicego, Mark E Olson, David S Gernandt, Tommaso Anfodillo

**Affiliations:** Department Territorio e Sistemi Agro-Forestali, Università degli Studi di Padova, Viale dell’Università 16, Legnaro, PD 35020, Italy; Instituto de Biología, Universidad Nacional Autónoma de México, Tercer Circuito Exterior s/n, Ciudad Universitaria, Ciudad de México 04510, Mexico; Instituto de Biología, Universidad Nacional Autónoma de México, Tercer Circuito Exterior s/n, Ciudad Universitaria, Ciudad de México 04510, Mexico; Department Territorio e Sistemi Agro-Forestali, Università degli Studi di Padova, Viale dell’Università 16, Legnaro, PD 35020, Italy

**Keywords:** Tracheids, conduit widening, drought resistance, tree height, leaf conductance, allometry, *Pinus*

## Abstract

**Background and Aims:**

In conifers, leaf length exhibits remarkable variation across and within species, even within the same individual. Leaves are often shorter in drier sites and at the tops of taller trees. Several hypotheses have been proposed to explain this shortening, but a clear causal framework is lacking. We hypothesize that conifer needles should exhibit a low rate of tip-to-base conduit widening leading to higher hydraulic resistance in long needles, explaining adaptive leaf shortening.

**Methods:**

We sampled needles from 22 *Pinus* species and one *Sequoia sempervirens* across a range of environmental conditions. We conducted a detailed intraspecific analysis on four *Pinus* species by measuring tracheid diameter along the needle, and an interspecific comparison by measuring tracheid diameter at the needle base across all species. In both analyses, we fitted tracheid diameter against distance from the needle tip and calculated the slope (*b*) of tip-to-base tracheid widening.

**Key Results:**

A low mean intraspecific widening slope (*b* = 0.12) was found, indicating that tracheid diameter increases only slightly from tip to base. This low widening rate cannot fully compensate for the increase in hydraulic resistance, which therefore increases with needle length. The interspecific slope of mean tracheid diameter at the needle base vs. needle length (0.25) was higher than the intraspecific mean, suggesting that longer-needled species may have wider conduits at the needle apex, offsetting needle length-imposed resistance.

**Conclusions:**

Our findings suggest that shorter needles should reduce hydraulic resistance under dry conditions or with height growth, maintaining leaf-specific conductance. We offer a novel explanation for the commonly observed pattern of needle shortening, interpreting it as an adaptive response rather than a physiological limitation.

## INTRODUCTION

Leaf size in plants shows remarkable interspecific variability worldwide, with leaf area varying by more than 100 000-fold and leaf length ranging from sub-millimetre to over 25 m in certain Arecaceae (Hallé, 1997; Dransfield *et al*., 2008; Kamga *et al*., 2018). In conifers, in addition to interspecific variability, leaf length shows high intraspecific plasticity between individuals and within the same individual, depending on climate, soil conditions, stand density and leaf position within the crown (Dobbertin *et al*., 2010; Chin and Sillett, 2016; Bert *et al*., 2021). It is widely reported that conifers produce shorter leaves on dry sites and during the driest years (Dougherty *et al*., 1994; Oleksyn *et al*., 1997; Cinnirella *et al*., 2002; Grill *et al.*, 2004; Zhang *et al*., 2011; Eimil-Fraga *et al*., 2015; Tyukavina *et al*., 2019). Moreover, at the tops of the tallest trees, leaves are much shorter than those on lower branches (Niinemets, 2002; Koch *et al*., 2004; Woodruff *et al*., 2004; Chin and Sillett, 2016; Chin *et al*., 2022).

Leaf shortening is often interpreted as a consequence of reduced cell turgor caused by water shortage, particularly during the elongation phase (Meinzer *et al*., 2008; Zhang *et al*., 2011; Eimil-Fraga *et al*., 2015). Similarly, several studies suggest that in taller plants, increased hydraulic resistance in the stem leads to lower cell turgor and, consequently, the production of shorter leaves (James *et al*., 2003; Koch *et al*., 2004; Woodruff *et al*., 2004; Woodruff and Meinzer, 2011). Most studies that have focused on leaf shortening in conifers conclude that lower water availability generally leads to a decrease in average turgor potential, which in turn limits the ability of leaves to expand during the elongation phase (Woodruff *et al*., 2004). These scenarios imply the assumption that longer leaves are developmentally impossible in a given situation, but that longer leaves would be favoured if they could be produced. From this point of view, low turgor conditions impose inevitable developmental reductions in leaf size, but the reason why trees with shorter leaves might have higher fitness compared to conspecifics with longer leaves remains largely unexplained.

It is well known that at the community level, plants have shorter leaves and a lower leaf area index (LAI) on dry sites (Chapin *et al*., 2011). Under resource-limited conditions, LAI decreases, which aligns with a reduction in overall evapotranspiration at the community scale. At the individual level, it could be assumed that shorter leaves would also lead to a lower total leaf area for a given body biomass. However, there is evidence that allometric relationships between plant organs (i.e. leaf mass vs. body mass) are actively maintained constant via plastic adjustments. Under conditions of severe water deficit, plants tend to produce shorter leaves, but total leaf mass for a given body mass remains relatively constant or slightly increases (Anfodillo *et al*., 2016; Kiorapostolou *et al*., 2018, 2020). This would suggest that even though leaves become shorter under lower water availability, total leaf mass relative to body mass, and thus water loss per unit body mass, remains relatively constant. Therefore, leaf shortening may not be an adaptation that reduces transpiration, but instead reflects an adaptive strategy that offers other functional advantages under dry conditions. Here, we explore what this adaptive strategy might be.

In the context of the soil–plant–atmosphere continuum, water movement through the xylem can be modelled using Darcy’s law (Reid *et al*., 2005), according to which flow is directly proportional to the water potential difference between leaves and soil, and inversely proportional to overall hydraulic resistance (from root to leaf). Under water-deficit conditions, effective adaptive mechanisms may include, among others, either an increase in the water potential gradient, for instance by decreasing the osmotic potential of the leaves (Badalotti *et al*., 2000; Jones, 2013), or structural modifications of leaf anatomy that reduce total hydraulic resistance, for instance wider xylem conduits, reductions in the overall length of the xylem hydraulic path or reduction of leaf mesophyll conductivity (Brodribb and Feild, 2010; Sack and Scoffoni, 2013).

Hydraulic resistance along the leaf water transport pathway arises from three main sources: the xylem conduits themselves, the thickness of the mesophyll (which determines the distance between xylem and sites of evaporation) and the conductance of the mesophyll tissue (Brodribb and Feild, 2010; Trueba *et al*., 2022). Key components include the resistance of xylem conduits, the thickness of the leaf mesophyll (i.e. the distance between xylem conduits and sites of evaporation), mesophyll conductance and the proportions of vascular to non-vascular tissue (Trueba *et al*., 2022). Importantly, these three components are expected to vary in a coordinated manner (Sack and Scoffoni, 2013). That is, if xylem resistance is relatively low, mesophyll resistance must also be low; otherwise, reducing only one component would offer no hydraulic advantage, as the unchanged components would still impose a bottleneck to water flow. This coordination leads to a remarkable invariance in the ratio between xylem and extraxylary (i.e. mesophyll) resistance (Scoffoni *et al*., 2012). Thus, analysing the factors driving variation in xylem conduit resistance can provide a basis for testing potential mechanisms that confer a selective advantage to plants in environments with limited water availability.

One of the most efficient anatomical strategies that reduces the hydraulic resistance of the entire conductive path is tip-to-base conduit widening. Conduit diameters, from the terminal leaf veins to the roots, widen approximating a power law (Anfodillo *et al*., 2006; Olson and Rosell, 2013; Rosell *et al*., 2017; Koçillari *et al*., 2021; Olson *et al*., 2021), as follows:


$$D=a\cdot L^{b}$$

where *D* is mean conduit diameter at a given distance *L* from the apex, *a* is apical conduit diameter and *b* is the widening rate. It is also well known that hydraulic resistance decreases with increasing conduit diameter (Tyree and Ewers, 1991; Vogel, 1994). The value of the exponent *b* is therefore critical in determining the degree to which this widening compensates for resistance associated with a certain plant height or leaf length. An exponent of about 0.20 or lower, as typically observed in conifer stems (Anfodillo *et al*., 2006, 2016; Koçillari *et al*., 2021; Zambonini *et al*., 2024), implies that as the hydraulic path increases in length, resistance is only partially compensated for (Williams *et al*., 2019). Conversely, values above 0.4, as reported in all angiosperm leaves studied to date (Coomes *et al*., 2008; Sack *et al*., 2012; Lechthaler *et al*., 2019; Levionnois *et al*., 2020), result in a steeper basipetal increase in diameter, which leads to lower hydraulic resistance, virtually independent of path length (Becker *et al*., 2000).

The narrowest conduits in a plant’s conductive system are typically found in the distal leaf veins. In conifers studied so far, ∼85 % of the total root-to-leaf hydraulic resistance (i.e. xylary and extraxylary resistance) is located within the leaves themselves, while in angiosperms, due to the high widening rate, the proportion of total resistance confined to the leaves is even higher, as much as 95 % (Lechthaler *et al*., 2020). These percentages highlight the critical role of leaves in determining the hydraulic resistance of the entire xylem pathway and, consequently, in influencing the ability of plants to meet hydraulic challenges such as drought or increasing height.

Given the close relationship between conduit diameter and resistance, it is essential to quantify how conduit diameter varies along leaves in conifers, and whether tip-to-base widening occurs. If so, it is important to estimate the value of the widening exponent *b*. The limited studies available on diameter variation along conifer leaves suggest that conduit diameter either remains relatively constant (Lechthaler *et al*., 2020), is higher in the middle portion (Jankowski *et al*., 2021) or is somewhat narrower at the apex, indicating modest widening (Gao *et al*., 2023).

Considering the high leaf length plasticity observed in conifers, where leaf length tends to decrease under low water availability and with height growth, we predict that the widening exponent in conifer leaves should be relatively low (*b* << 0.20). Given a low widening rate, a reduction in needle length would lead to a substantial decrease in hydraulic resistance. Our hypothesis is that individuals with shorter leaves should have lower overall hydraulic resistance (from roots to leaves), resulting in greater fitness under water-deficit conditions compared to conspecifics with longer leaves. For example, using Poiseuille’s law and considering a single conduit with an apical diameter of 5 µm and a widening rate of 0.15, reducing its length from 4 to 1 cm results in a decrease in leaf hydraulic resistance of more than 60 % (Anfodillo and Olson, 2024).

To examine the hypothesis of low tip-to-base tracheid widening in pine needles, we combined across-species and within-species sampling. We conducted a detailed analysis of the longitudinal profile of xylem conduit diameter in four species of the genus *Pinus*. These four species are very closely related, making up the *devoniana* clade, and yet span practically the entire range of needle length in *Pinus*. In addition, to test the generality of tip-to-base conduit widening across pines, and whether or not apical needle tracheid diameter remains invariant with leaf length (Sack *et al*., 2012), we conducted a comparative analysis across 22 species of *Pinus* plus *Sequoia*, measuring conduit diameter at the base of needles with lengths ranging from 13 to 451 mm.

## MATERIALS AND METHODS

### Samplings and experimental design

To analyse the broadest possible range of needle lengths in conifers, we chose the genus *Pinus*, one of the most species-rich conifer genera and the one exhibiting the widest variation in leaf length, from less than 1 cm to nearly 50 cm (Farjon, 2010; Wang *et al*., 2019). We sampled *Pinus* species in Mexico, which is the richest centre of morphological and species diversity for the genus (Farjon, 1996; Gernandt and Pérez-de la Rosa, 2014; Gauthier *et al*., 2015; Sáenz-Ceja *et al*., 2022). Some samples were collected from living trees, while others were obtained from the National Herbarium of Mexico (MEXU) at the Instituto de Biología, Universidad Nacional Autónoma de México (UNAM), and rehydrated prior to anatomical analysis (Table 1). In total, 22 *Pinus* species were sampled. One species, *Pinus hartwegii*, was collected at two different sites due to the marked difference in needle length observed between them: one sample was from a moist site in the state of Hidalgo (hereafter referred to as *Pinus hartwegii_WET_*), and the other from a much drier, high-elevation site (4000 m a.s.l.) on the Nevado de Toluca volcano in Mexico State (hereafter *Pinus hartwegii_DRY_*). Given the potential relevance of our findings for tall-growing species, a sample of *Sequoia sempervirens* was also included to obtain preliminary data on this species. Needle samples from living trees were collected from low branches, using a pole pruner when necessary.

**Table 1.** List of species included in the study, ordered by the length of the analysed leaf.

Sample no.	Species	Leaf length (mm)	Fresh/herbarium
**1**	*Pinus ayacahuite*	451	Fresh
**2**	Pinus devoniana*	339	Fresh
**3**	*Pinus patula*	214	Fresh
**4**	Pinus montezumae*	210	Herbarium
**5**	***Pinus hartwegii*** *_WET_* *	197	Fresh
**6**	Pinus pseudostrobus*	185	Fresh
**7**	*Pinus maximartinezii*	125	Fresh
**8**	*Pinus teocote*	112	Fresh
**9**	*Pinus halepensis*	110	Fresh
**10**	*Pinus rzedowskii*	95	Herbarium
**11**	*Pinus nelsonii*	78	Herbarium
**12**	*Pinus pinceana*	71	Herbarium
**13**	*Pinus lagunae*	64	Herbarium
**14**	*Pinus johannis*	53	Herbarium
**15**	*Pinus rigida*	49	Fresh
**16**	*Pinus culminicola*	46	Herbarium
**17**	*Pinus remota*	33	Herbarium
**18**	*Pinus discolor*	33	Herbarium
**19**	***Pinus hartwegii*** *_DRY_* *	30	Fresh
**20**	Sequoia sempervirens*	28	Fresh
**21**	*Pinus cembroides*	26	Herbarium
**22**	*Pinus quadrifolia*	19	Herbarium
**23**	*Pinus californiarum*	18	Herbarium
**24**	*Pinus monophylla*	13	Herbarium

For each sample, it is indicated whether it was taken from a living specimen (‘Fresh’) or from the National Herbarium of Mexico (‘Herbarium’). For all species, an anatomical cross-section was taken 2 mm from the leaf base. For species highlighted in bold and marked with an asterisk (*), cross-sections were taken along the entire longitudinal profile. For *Pinus hartwegii*, the two samples are distinguished by a suffix indicating the respective site water availability: sample no. 5 is referred to as *Pinus hartwegii_WET_* and sample no. 19 as *Pinus hartwegii_DRY_*.

**Fig. 1.**
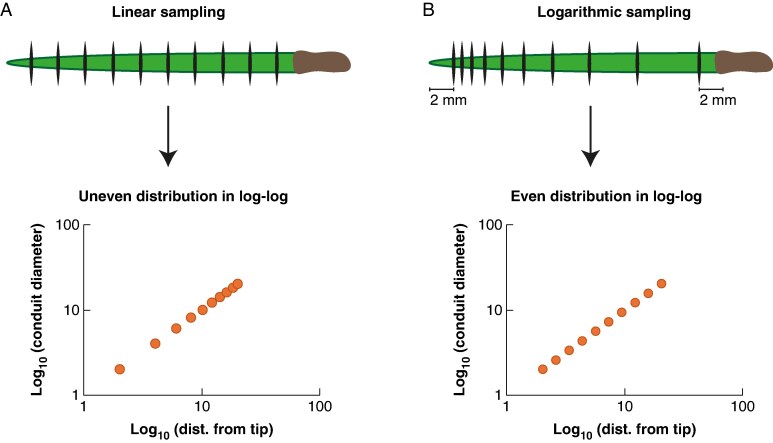
Needle sectioned at 10 points at different distances from the tip, with vertical black lines indicating the sectioning points. (A) If the sectioning points are distributed linearly along the needle, they appear unevenly distributed in a bi-logarithmic plot after the necessary logarithmic transformation, with fewer points at the lowest values where functional impacts of diameter changes are most significant. (B) Sampling strategy used in this study: sectioning points were distributed according to a logarithmic scale, with closer spacing near the apex and increasing spacing toward the base. This results in data points that are evenly distributed in a log–log regression. The first and last sections were taken 2 mm from the needle tip and base, respectively. The fascicle sheath was removed if necessary.

Many studies have measured mean tracheid diameter in conifer needles, but typically at a single point along the needle and often without taking into account and reporting the distance from the apex. However, to estimate the rate of tracheid widening, diameter measurements must be paired with their corresponding distance from the needle tip. To obtain detailed insights into intraspecific widening, we analysed anatomical longitudinal profiles within the needles of five different samples (Table 1, species in bold with an asterisk), taking cross-sections at increasing distances from the needle tip and measuring the mean tracheid diameter at each point (Fig. 1B). In pine species we took from eight to 12 cross-sections per needle in relation to needle length. The four pine species used for longitudinal needle profiles were chosen because they are very close phylogenetic relatives (Hernández-León *et al*., 2013, Jin *et al*., 2021, Willyard *et al*., 2021), and yet span virtually the entire range of mature needle lengths observed across the entire genus (Farjon, 2010; Wang *et al*., 2019). The wide range of needle lengths among the species sampled for tip-to-base tracheid profiles – from 3 to 33.9 cm – allows us to test whether there is variation in apical tracheid conduit diameter or tip-to-base widening slope with needle length.

Since conduit diameter, like many other allometric traits, is expected to vary along the needle axis following a power law, a given change in conduit size has different functional implications depending on whether it occurs at the lower end (i.e. near the needle tip) or at the upper end (i.e. near the needle base) of the size range. For example, the same 5-µm increase in conduit diameter has a modest effect on hydraulic resistance (*R*_H_) if the change is from 50 to 55 µm – *R*_H_ decreases by a factor of 1.5 – but has a 10-fold greater impact if the diameter increases from 5 to 10 µm – *R*_H_ decreases by a factor of 16. In allometric contexts, logarithmic transformation is therefore crucial, as it expands the variation at lower values, so it weighs more where functional consequences are most significant, and compresses variation at higher values, where it is less functionally important (Kerkhoff and Enquist, 2007).

Properly defining the sampling points along the needle is therefore essential to obtain robust regressions that reflect hydraulic structure accurately. Cross-sections should be taken at distances from the apex that follow a logarithmic scale, i.e. more closely spaced near the tip and increasingly spaced towards the base (Olson *et al*., 2021). This ensures that data points are evenly distributed once the regression is performed in a log–log framework (Fig. 1B).

To test the notion that selection shapes conduit diameters along needles similarly across *Pinus*, we conducted a comparative analysis including 22 species drawn from across the pine phylogeny and a wide range of environments and geographical locations (Table 1). Since obtaining tracheid diameter variations along full longitudinal profiles is relatively time-consuming, we measured mean tracheid diameter at the needle base for our comparative sampling. If selection shapes tip-to-base tracheid widening in identical ways across species, and if apical needle tracheid diameter is identical across species, then both the slope and the intercept of the interspecific regression based on basal tracheid diameter would be expected to match the mean slope obtained from the intraspecific regressions of the longitudinal needle profiles. Conversely, if the interspecific slope differs from the mean of the intraspecific slopes, this indicates that either apical xylem conduit diameter or the widening rate varies among species.

### Anatomical analysis

For each species, one average-sized needle was selected to obtain anatomical cross-sections and measure tracheid diameter. Fresh samples were preserved in 70 % ethanol. Samples collected from the herbarium were rehydrated prior to anatomical analysis by boiling them for 10–12 min in a solution of water and a drop of dishwashing liquid (as a wetting agent), and then leaving them in water for ∼12 h or until they sank.

**Fig. 2.**
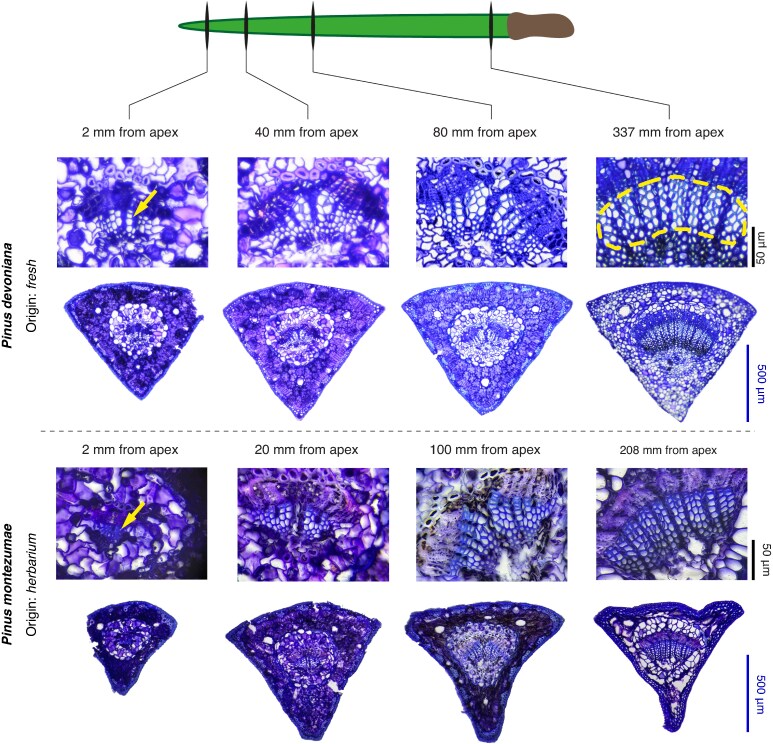
Examples of anatomical cross-sections along the longitudinal profile of the needle. A species sampled from a living individual (*Pinus devoniana*) and a species sampled from herbarium material, rehydrated prior to sectioning (*Pinus montezumae*), are shown. For each species, the apical section (2 mm from the tip), the basal section (2 mm from the needle base) and two intermediate sections are presented. All complete anatomical sections are shown at the same scale (blue bar = 500 µm), and magnified views are shown at the same scale (black bar = 50 µm). In the basal section of *P. devoniana*, the xylem conduit bundle is highlighted with a yellow dashed line. In all sections, the phloem is located just above the xylem. Notably, tracheids in the apical sections (yellow arrows) have larger diameters in the longer-needled species *P. devoniana* (339 mm) compared with the shorter-needled *P. montezumae* (210 mm), showing apical conduit widening as a compensatory mechanism for the increased hydraulic resistance associated with longer needle length.

Anatomical cross-sections of the needles (15–40 µm thick) were obtained without embedding, using a GSL1 sliding microtome (Gärtner *et al*., 2014) (Fig. 2).

To ensure a sufficient number of measurable tracheids, the first cross-section of each longitudinal profile was taken 2 mm from the needle apex. To avoid potential tracheid deformation near the needle base, which is often much less lignified than the rest of the needle, the final cross-section was taken 2 mm from the needle base (Fig. 1B), after removing the fascicle sheath if necessary. For the same reason, in the interspecific analysis, the basal cross-section was also taken 2 mm from the needle base.

Sections were stained with a solution of water and cresyl violet acetate, mounted on glass slides with a drop of water containing ∼5 % glycerol, and covered with a cover glass. Cross-sections were analysed under light microscopy (Velab VE-B7; 1000× magnification), and tracheid diameters were measured using the ocular micrometer. For each cross-section we measured at least 30 randomly selected tracheids and the mean tracheid diameter was calculated. We fitted the relationship between mean tracheid diameter and distance from the needle tip, implemented in GraphPad Prism 10 (GraphPad Software, LLC). The slope (*b* value) and the *y*-intercept (*a* value), representing tracheid diameter at 1 mm from the needle tip, were estimated with 95 % confidence intervals (CIs) using the profile likelihood method. Significant differences between parameters were inferred when their CIs did not overlap.

Our comparisons focused on the slopes of the relationships between mean conduit diameter at a certain cross-section and the distance of that section from the needle tip, both within needles and across species means. Herbarium sampling allowed us to include a broad range of *Pinus* species. To test whether preservation status (*Fresh* vs. *Herbarium*; Table 2) affected the scaling of base tracheid diameter with needle length, we fit a model predicting log_10_ mean tracheid diameter at the needle base as a function of log_10_ leaf length and a categorical variable indicating preservation status. The interaction term was not significant (*P* = 0.27), indicating that the scaling slope did not differ between fresh and herbarium specimens. We therefore re-fit the model without the interaction term. In this model, the preservation status was also not significant (*P* = 0.08), indicating no difference in tracheid diameter for a given needle length between *Fresh* and *Herbarium* samples. Consequently, we pooled the data and treated the two types of material equivalently.

**Table 2.** Values describing the linear regression fitting of tracheid (conduit) diameter along the needle in four *Pinus* species.

Species	Leaf length (mm)	*a* (µm)	*a* – 95 % CI	*b*	*b* – 95 % CI	*b –* Intersp. mean
***Pinus devoniana***	339	5.85	5.26–6.59	**0.11**	0.08–0.14	**0.12**
***Pinus montezumae***	210	4.56	3.99–5.39	**0.13**	0.08–0.17	
***Pinus hartwegii_WET_***	197	4.32	3.97–4.72	**0.15**	0.13–0.18	
***Pinus pseudostrobus***	185	4.69	4.06–5.47	**0.13**	0.08–0.17	
***Pinus hartwegii_DRY_***	30	4.13	3.68–4.73	**0.09**	0.04–0.14	
***Sequoia sempervirens***	28	5.37	5.35–5.40	0.045	0.042–0.048	

Preliminary results for *Sequoia sempervirens* are also included. Samples are ordered from the species with the longest to the shortest needle. The *y*-intercept (*a*) and slope (*b*) of the fitted curves are reported, along with their respective 95 % confidence intervals (CIs). The 95 % CI of the interspecific mean *b* value for the genus *Pinus* ranges from 0.094 to 0.15. No significant differences were found among the slopes of *Pinus* species.

## RESULTS

### Within-needle longitudinal analysis (intraspecific)

**Fig. 3.**
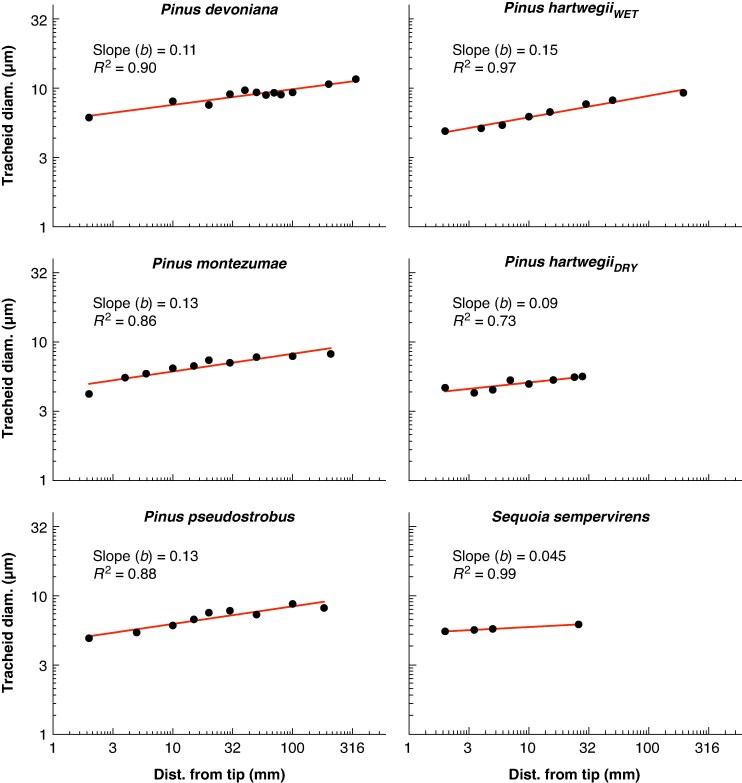
Linear regressions of mean tracheid diameter along the longitudinal profile of needles. Both axes are displayed on a logarithmic scale. The figure includes the four *Pinus* species for which full longitudinal profiles were measured (∼10 cross-sections per needle), along with preliminary results for *Sequoia sempervirens* (based on four cross-sections: one at 2 mm from the tip, one at 2 mm from the base and two at intermediate positions). For each species, the slope (*b*) and the coefficient of determination (*R*^2^) are reported. The 95 % confidence intervals for the slopes and intercepts are shown in Table 2.

**Fig. 4.**
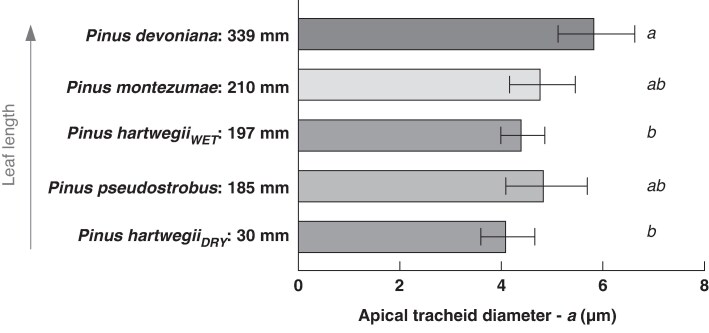
Longer needles appear to have wider tracheids at the needle tip. Intercept values (*a*) of the regressions for the four *Pinus* species for which a full longitudinal profile was measured are shown with their respective 95 % confidence intervals (CIs) as error bars. Letters (*a*, *b*) indicate significant differences based on non-overlapping CIs. The shortest-needled species (*Pinus hartwegii_DRY_*) had significantly narrower apical tracheids compared to the longest-needled species (*Pinus devoniana*). The intermediate needle-length species displayed intermediate apical tracheid diameters. These results suggest that apical tracheid diameter increases with needle length.

The range of needle lengths examined across the four *Pinus* species selected for measuring longitudinal, within-needle, profiles of tracheid diameter ranged from 3.0 cm in *Pinus hartwegii_DRY_* to 33.9 cm in *Pinus devoniana*. Mean tracheid diameter increased from the tip to the base of the needle closely following a power law (i.e. linear regression in log–log), with the distance from the tip explaining between 73 and 97 % of the variability in mean tracheid diameter (Fig. 3). Values of the widening rate (*b*, the slope of the regression) among the pine species fell within a narrow range, from 0.09 in *Pinus hartwegii_DRY_* to 0.15 in *Pinus hartwegii_WET_*, with an overall average of 0.12 (Table 2). No statistically significant differences in the widening slope (*b*) were observed among the four *Pinus* species (Table 2). However, the *y*-intercept of the regression (*a* value), which represents tracheid diameter at 1 mm from the needle tip, differed among species. *Pinus devoniana*, which had the longest needles, had a significantly higher *a* value compared to both samples of *Pinus hartwegii*, which did not differ from each other despite their substantial difference in needle length. The other two species, *Pinus pseudostrobus* and *Pinus montezumae*, had apical conduit diameters (*a* values) that did not differ significantly from the other species (Table 2 and Fig. 4). The widening rate in *Sequoia sempervirens* appears to be even lower, with a slope of 0.045, and the diameter of apical conduits appears to be relatively wide compared to the *Pinus* species (Table 2).

### Among-needles analysis (interspecific)

**Fig. 5.**
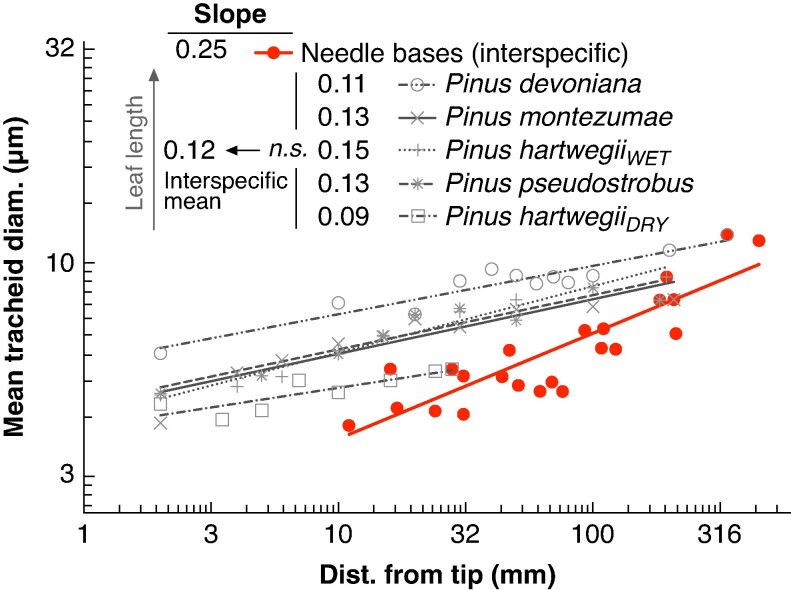
Linear regression of mean tracheid diameter at the base of the needles of 22 *Pinus* species plotted against needle length (interspecific relationship, red line). Both axes are on a logarithmic scale. The slope (*b*) of the fitted curve was 0.25 (*R*^2^ = 0.74). The 95 % confidence intervals are: slope (*b*), 0.19–0.30; intercept (*a*), 1.75–2.20. The slopes of the intraspecific needle profiles (black lines) did not differ, with a mean value of 0.12.

The interspecific analysis included 22 *Pinus* species, with needle lengths ranging from 1.3 to 45.1 cm. Mean tracheid diameter at the needle base increased with increasing distance from the tip (i.e. needle length), with a slope of 0.25 (Fig. 5). The interspecific slope was significantly higher than the slopes obtained in the intraspecific analysis (Table 2).

## DISCUSSION

Despite xylem anatomy in conifer leaves being extensively studied, particularly within the genus *Pinus* (Trueba *et al*., 2022), information on hydraulic architecture along the needle axis remains limited (Zwieniecki *et al*., 2006; Wang *et al*., 2019; Lechthaler *et al*., 2020; Jankowski *et al*., 2021; Gao *et al*., 2023). Specifically, although tracheid diameter plays a crucial role in determining the overall hydraulic resistance of a plant, few studies have investigated variation in tracheid diameter along the lengths of conifer leaves, and the available findings are often inconsistent (Gebauer, 2024). To our knowledge, no studies have specifically focused on tip-to-base widening in conifer leaves, a mechanism that has been demonstrated to be both universal across the stems of terrestrial plants and effective in reducing hydraulic resistance. This study represents the first systematic attempt to quantify tip-to-base widening in conifer leaves. The results confirm our expectation that widening rates along the lengths of conifer leaves is generally low (<0.20). We discuss the implications of our findings for explanations of the marked variation in leaf length within and across conifer species.

### Longitudinal analysis – low intraspecific widening

**Fig. 6.**
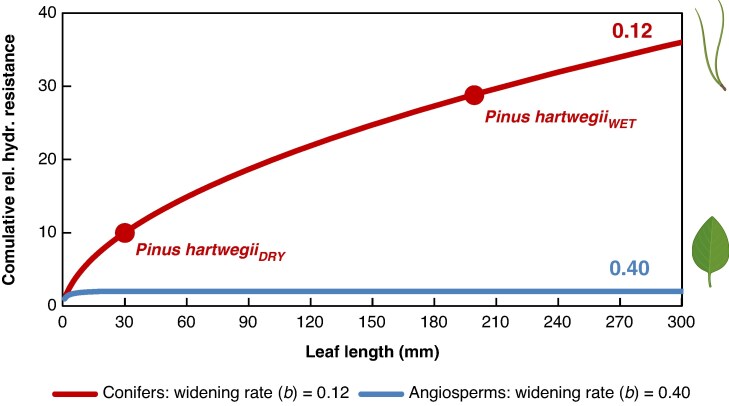
Model of cumulative relative hydraulic resistance (i.e. resistance relative to the value at 1 mm from the tip) along the entire leaf path under different xylem conduit widening rates (slopes of the tracheid basipetal widening regressions; *b* values). For angiosperms (blue line), a widening rate of 0.40 was used, resulting in virtually constant resistance across different leaf lengths. For conifers (red line), a widening rate of 0.12 was used; in this scenario, the hydraulic resistance of a 30-mm needle (as in *Pinus hartwegii_DRY_*) is 2.8 times lower than that of a 197-mm needle (as in *Pinus hartwegii_WET_*), highlighting a clear functional advantage under low water availability.

The low rate of tip-to-base tracheid widening supports our hypothesis that variation in needle length represents a key mechanism maintaining leaf-specific conductance across gradients in soil water potential and plant height. The four pine species in which we measured tracheid diameter profiles along the needle axis showed strikingly similar values for the widening slope (*b*). Across all tip-to-base profiles, the mean value of *b* was 0.12, with no statistically significant differences among species (Table 2 and Fig. 3). This consistently low widening rate has important implications for water transport and the coordination of structure and function in pine needles. With their low conduit widening rate, all else being equal (such as needle tip ‘starting’ tracheid diameter), longer needles have substantially higher total hydraulic resistance (Fig. 6), which reduces conductance to the needle and lowers photosynthetic capacity.

The anatomical characteristics measured allow calculation of the theoretical hydraulic resistance of a conduit, for example in the needles of two *Pinus hartwegii* individuals (Fig. 6). Using the average apical conduit diameter (parameter *a*, 4.23 µm; the two individuals did not differ), mean widening rate (parameter *b*, 0.12) and total needle lengths (Table 2), overall hydraulic resistance of the longest needle (197 mm) is ∼2.8 times higher than that of the shortest one (30 mm). This implies that, all else being equal (e.g. the same water potential gradient between leaf and soil), the water flow to longer needles would be reduced by a factor of 2.8 compared to the potential flow in shorter needles (Fig. 6). This reduction in resistance is particularly significant because the distal portions of the whole hydraulic path have xylem elements with the narrowest diameters, which contribute disproportionately to the overall resistance (Lechthaler *et al*., 2020).

In angiosperm leaves, tip-to-base xylem conduit widening has been reported to be substantially greater compared to conifers, with widening rates ranging from 0.33 to 0.55, with a mean of around 0.40 (Coomes *et al*., 2008; Lechthaler *et al*., 2019; Levionnois *et al*., 2020, 2021). This much higher rate of conduit widening concentrates hydraulic resistance toward the very tip of the leaf, where conduits are narrowest, while the remaining portion of the path adds only minimal resistance due to the marked increase in conduit diameter (Lechthaler *et al*., 2020). As a result, in angiosperms, leaf hydraulic resistance is almost invariant with leaf length (Fig. 6). This causal decoupling between leaf length and hydraulic resistance makes it possible, for example, in certain Arecaceae, for leaves to grow to over 25 m in length (Hallé, 1997; Dransfield *et al*., 2008; Kamga *et al*., 2018).

This also helps explain why, in some beech forests with markedly different water availability, no reduction in leaf size was observed at sites with higher water deficit; on the contrary, an increase in average leaf length was reported (Meier and Leuschner, 2008). An increase in leaf size under conditions of severe summer drought would pose a significant disadvantage if the leaf conduit widening rate were as low as that observed in conifers (0.12), because it would lead to a substantial increase in total leaf hydraulic resistance. However, in vessel-bearing angiosperm species, consistently high widening rates (about 0.4–0.5) have been measured, resulting in hydraulic resistance that remains largely independent of leaf length (Fig. 6). As a consequence, leaf length in angiosperms does not appear to be a trait that significantly affects plant resistance to drought (Homeier *et al*., 2021).

The same line of reasoning can be applied to explain the variation in leaf size observed in very tall trees, as in *Sequoia sempervirens*, which is well known for its high leaf length variation, with much shorter needles at greater crown heights (Koch *et al*., 2004). Our preliminary results indicate that the leaf xylem of *Sequoia sempervirens* exhibits an even lower widening rate, with a slope of 0.045, one of the lowest tip-to-base widening values ever recorded. These findings are consistent with the hypothesis that leaf shortening is an adaptive mechanism that reduces hydraulic resistance along hydraulic pathways in conifers, such as those found at the tops of tall trees. All else being equal, assuming an apical conduit diameter of 5.37 µm (parameter *a*, Table 2) and a widening rate of 0.045, a leaf length of 2.8 cm compared to a leaf length of 0.5 cm, consistent with observations on branches from taller trees (Koch *et al*., 2004), would result in a more than 4-fold decrease in total hydraulic resistance.

Turgor variation is often implied as being the main cause of changes in leaf length (Koch *et al*., 2004; Meinzer *et al*., 2008; Zhang *et al*., 2011; Eimil-Fraga *et al*., 2015). However, we propose that turgor represents only the proximate cause (i.e. the mechanism by which leaf size is reduced), not the ultimate cause (i.e. why plants with smaller leaves at the top of the canopy are favoured by natural selection). Our view predicts that both longer and shorter leaves should be developmentally possible but that selection favours certain leaf lengths at certain positions in the canopy. From this point of view, the fitness of plants with smaller leaves at the top of the canopy is greater, as this morphological adjustment significantly reduces overall hydraulic resistance, thereby helping to maintain water flow to the leaves despite the longer hydraulic path.

Our results would support the notion of ‘sectoriality’ in plant hydraulic systems. Sectoriality is the idea that, even though plant conduits are highly interconnected, in functional conductive systems, some conduits preferentially supply some leaves while other conduits preferentially supply others (Orians *et al*., 2005; Zanne *et al*., 2006; David *et al*., 2012). So, instead of all conduits somehow supplying all leaves, conduits on one side of a tree would preferentially supply leaves on that side (Salguero-Gómez and Casper, 2011). Relevant to our discussion here is that it suggests that some conduits should preferentially supply leaves low in the tree, and other conduits leaves high in the tree. One interpretation of sectoriality implies that at the base of the tree, conduits supplying leaves low in the tree should be narrower than those high in the tree. If conduits were all of the same diameter at the base, then the conduits supplying shorter path lengths would have lower resistance and therefore water would preferentially flow to lower leaves and less to higher ones. Narrower stem conduits leading to shorter pathways would represent a mechanism equalizing conductance to each unit of leaf area regardless of distance from the base. In addition, our results suggest that leaf length could be involved in this regulation of conductance to the entire crown. If longer leaves of higher resistance are deployed at the apices of shorter path lengths, with shorter leaves of much lower resistance at the apices of longer path lengths, then leaf resistance would contribute to equalizing conductance per unit leaf area throughout the crown. This pronounced sectoriality aligns with the hydraulic architecture proposed by West *et al*. (1999), which suggests a one-to-one relationship between conduits and leaves. In contrast, alternative models, such as that of Savage *et al*. (2010), which describe extensive conduit coalescence toward the base of the stem, resulting in individual conduits supplying multiple leaves, imply reduced sectoriality and currently appear to have less empirical support.

### Increase in apical tracheid diameter as a possible compensatory mechanism

Our across-species analysis suggests a potential structural compensation that accompanies an increase in needle length and helps maintain conductance constant. The slope of the interspecific relationship was about twice the intraspecific one, 0.25 (Fig. 5) compared to 0.12 (Table 2). Comparable results have been found in Wang *et al.* (2019), on four *Pinus* species, although the regression slope was not explicitly reported. If apical needle tracheid diameter (*a*) and tip-to-base widening rate were the same across species, then the interspecific and intraspecific slopes would be identical. The much higher across-species slope implies one of two scenarios. The first is that longer needles have higher tip-to-base tracheid widening rates. Given that we did not observe any such variation across the four species whose tip-to-base widening rates we examined directly (Table 2), and that they spanned a very wide range of needle lengths, this possibility seems highly implausible. It is more likely that the apical tracheid diameters, i.e. the ‘starting’ diameters at the apexes of the needles, are wider. Our results suggest an increase of apical tracheid diameter with needle length (Figs 4 and 5) Broader sampling is needed to confirm this possible relationship.

Given a similar tip-to-base tracheid widening rate within needles across species, wider tracheids at the needle tip mean wider tracheids throughout the entire needle. Wider tracheids throughout dramatically reduce resistance. So, wider conduits at the apex and throughout a longer needle would be a mechanism beyond just tip-to-base widening that maintains conductance constant per unit leaf area (Anfodillo and Olson, 2024). The effectiveness of the compensatory mechanism involving conduit widening at the needle apex can be assessed by calculating the total hydraulic resistance of the entire needle across different species. Using empirical anatomical data, specifically needle length, apical conduit diameter and widening rate (Table 2) for *Pinus devoniana* and *Pinus hartwegii_DRY_* (the species with the longest and shortest needles, respectively), permits estimation of the cumulative resistance. The results show that total hydraulic resistance is similar, differing by only about 15 %, but slightly higher in *Pinus hartwegii_DRY_* despite a more than 10-fold difference in needle length.

In the absence of any compensatory widening mechanism at the apex (i.e. by setting for *Pinus devoniana* the apical conduit diameter in the model to 4.13 µm, as in *Pinus hartwegii_DRY_*), the overall resistance of *Pinus devoniana* needles would be ∼3.5 times higher. Selection would not be expected to favour such a drastic increase in resistance, given that these would be associated with commensurately drastic decreases in photosynthetic rate.

Analogous apical conduit widening appears to occur in vessel-bearing angiosperm stem vessels; terminal twig vessel diameters are wider with height growth (Zach *et al*., 2010; Olson *et al*., 2014, 2018, 2020; Echeverría *et al*., 2019). Mechanisms that further reduce hydraulic resistance beyond tip-to-base conduit widening are known as ultra-widening permeability (Anfodillo and Olson, 2024), and are probably crucial, though often overlooked, in maintaining conductance to leaves as plants grow taller and leaves elongate. In angiosperm leaves, the high widening rate of ∼0.40 (Coomes *et al*., 2008; Lechthaler *et al*., 2019; Levionnois *et al*., 2020) can result in very wide vessels at the petiole level, over 80 µm in diameter for example (Levionnois *et al*., 2020), which essentially fully compensates for increased leaf length (Fig. 6). This eliminates the need for additional compensatory mechanisms beyond the tip-to-base widening itself. In conifer leaves, instead, selection seems to have favoured a hydraulic structure in which tracheid diameters remain relatively narrow: in our dataset, the maximum diameter at the needle base is ∼10 µm. This results in a low tip-to-base rate of conduit widening in conifer leaves (a mean of 0.12 for the pines in this study) and suggests that additional ultra-widening mechanisms, such as the apical conduit widening described here, may be required to counteract the increased hydraulic resistance associated with leaf elongation (Fig. 6).

The idea that tracheids widen relatively little is further supported by the observation that, even at the base of the tallest conifers on Earth, tracheid diameters remain modest. Basipetal tracheid widening appears to reach an asymptotic maximum of ∼50 µm, beyond which further increases do not occur (Williams *et al*., 2019). Conduit widening itself is not likely to be sufficient to fully explain mechanisms compensating for hydraulic resistance increases in the tallest trees, but many other traits should be studied, such as variation in the number of conduits (Echeverría *et al*., 2019), the role of the extraxylary pathway within the leaf (Zwieniecki *et al*., 2004, 2007; Trueba *et al*., 2022; Scoffoni *et al*., 2023), differences in xylem permeability other than widening (Anfodillo and Olson, 2024), the possible contribution of foliar water absorption (Limm *et al*., 2009; Ishii *et al*., 2014; Kerhoulas *et al*., 2020, Chin *et al*., 2022) and, as our results suggest, the role of leaf length variation.

## CONCLUSIONS

Our findings shed light on the functional anatomy of conifer needles, providing insight into their adaptive plastic responses to environmental challenges. This study offers a novel perspective on the significance of shorter needle length observed in conifers in dry environments and in the tallest trees (i.e. redwoods), as an adaptive response to low water availability that allows for reduced leaf hydraulic resistance.
